# A broad-spectrum antibiotic, DCAP, reduces uropathogenic *Escherichia coli* infection and enhances vorinostat anticancer activity by modulating autophagy

**DOI:** 10.1038/s41419-018-0786-4

**Published:** 2018-07-13

**Authors:** Giulia Allavena, Doriana Debellis, Roberto Marotta, Chetanchandra S. Joshi, Indira U. Mysorekar, Benedetto Grimaldi

**Affiliations:** 1 Laboratory of Molecular Medicine, Fondazione Istituto Italiano di Tecnologia, via Morego 30, 16163 Genova, Italy; 2 Electron Microscopy facility, Fondazione Istituto Italiano di Tecnologia, via Morego 30, 16163 Genova, Italy; 3 Department of Obstetrics & Gynecology, Washington University School of Medicine, St. Louis, MO 63110 USA; 4 Centre for Reproductive Health Sciences, Washington University School of Medicine, St. Louis, MO 63110 USA

## Abstract

The cellular recycling pathway of autophagy plays a fundamental role in adaptive responses to nutrient deprivation and other forms of stress under physiological and pathological conditions. However, autophagy can also be a double-edge sword during certain bacterial infections (such as urinary tract infections) and in cancer, where it can be hijacked by the pathogens and cancer cells, respectively, to promote their own survival. Thus, autophagy modulation can potentially have multiple effects in multiple contexts and this property can be leveraged to improve outcomes. In this report, we identify that a broad-spectrum antibiotic, 2-((3-(3, 6-dichloro-9H-carbazol-9-yl)-2-hydroxypropyl) amino)-2-(hydroxymethyl) propane-1, 3-diol (DCAP) modulates autophagy. We employed combined biochemical, fluorescence microscopy and correlative light electron microscopy approaches to demonstrate that DCAP treatment blocks autophagy at the late stages by preventing autophagolysosome maturation and interrupting the autophagic flux. We further show that, DCAP significantly reduces UPEC infection in urinary tract epithelial cells via inhibition of autophagy. Finally, we reveal that DCAP enhances the anticancer activity of the histone acetyltransferase (HDAC) inhibitor, vorinostat, which has been reported to increase susceptibility to bacterial infections as a common adverse effect. Collectively, our data support the concept that DCAP represents a valuable chemical scaffold for the development of an innovative class of bactericidal autophagy inhibitors for treatment of urinary tract infections and/or for adjuvant therapy in cancer treatment.

## Introduction

Macroautophagy (henceforth referred to as autophagy) is a proteosomal-independent degradative mechanism that promotes catabolism and recycling of diverse cytoplasmic content^1,2^. A number of autophagy-related genes (ATGs) are implicated in the formation and maturation of cytoplasmic double membrane vesicles, named autophagosomes, which engulf a variety of macromolecules and organelles. Mature autophagosomes fuse with lysosomes to form digestive acidic vescicles, called autophagolysosomes or autolysosomes and contents are degraded and recycled^3–5^.

A key autophagy gene/protein component is the microtubule Associated Protein 1 Light Chain 3 (LC3), lipid-conjugated LC3 is specifically recruited to the autophagosomal membranes, assisting both formation and maturation of autophagosomes^3^. Accordingly, the evaluation of lipid-modified LC3 and/or its cellular localization serves as a suitable system to monitor autophagosome turnover under diverse conditions^6,7^. In addition to ATGs, scaffolding/adaptor proteins like p62 also known as sequestosome 1 (SQSTM1) assist the recruitment of specific targets to the autophagomes. p62/SQSTM1, serves as a link between LC3 and ubiquitinated substrates^8^. SQSTM1 and SQSTM1-bound polyubiquitinated proteins are incorporated into the completed autophagosome and are degraded in autolysosomes^9^.

Accumulating evidence indicates that autophagy is a fundamental adaptive response to starvation and other forms of stress^10–12^, in tissue homeostasis^13^, cellular differentiation and development^14^, and ageing^15,16^. Thus, pharmacological induction of autophagy has been proposed for preventing the development of human pathologies or for reversing the adverse effects of ageing^17^.

Nonetheless, autophagy can also be detrimental as is the case with certain pathogens that co-opt the pathway for their survival. For example, uropathogenic *Escherichia* *coli* (UPEC), the predominant cause of Urinary Tract Infections (UTIs), can persist within the urinary bladder epithelium (urothelium) by hijacking the autophagy pathway, forming quiescent intracellular reservoirs within autophagosomes wherein they are refractory to antibiotic treatment^18,19^.

On the other hand, in case of cancer, established tumor cells often enhance autophagic flux to increase their survival under limited nutrition conditions and/or to overcome chemotherapy induced stress by recycling cellular components to produce alternative sources of energy.^20–22^. Consequently, a block of autophagy has been proposed as a suitable strategy for improving the anticancer activity of several neoplastic agents^21^. As an example, genetic or pharmacological inhibition of autophagy in breast cancer cells improved the anticancer activity of the histone de-acetyltransferase (HDAC) inhibitor, vorinostat^7,23^.

Here we show that a recently identified broad-spectrum antibiotic, DCAP (2-((3-(3, 6-dichloro-9H-carbazol-9-yl)-2-hydroxypropyl) amino)-2-(hydroxymethyl) propane-1, 3-diol), can inhibit canonical autophagy in human cancer cells by blocking autophagic flux and preventing maturation of autophagolysosomes. Further, DCAP-mediated autophagy blockade limits UPEC infection of bladder cells. Finally, DCAP enhances the anticancer activity of vorinostat against breast cancer cells. Thus, we propose DCAP as a valuable chemical scaffold for the development of antibacterial, anti-autophagic drugs to treat UTIs and for use as combination therapy agents along with vorinostat in breast cancer treatment.

## Results

### DCAP, a broad-spectrum antibiotic, negatively modulates autophagy and inhibits autophagic degradation

**Fig. 1:**
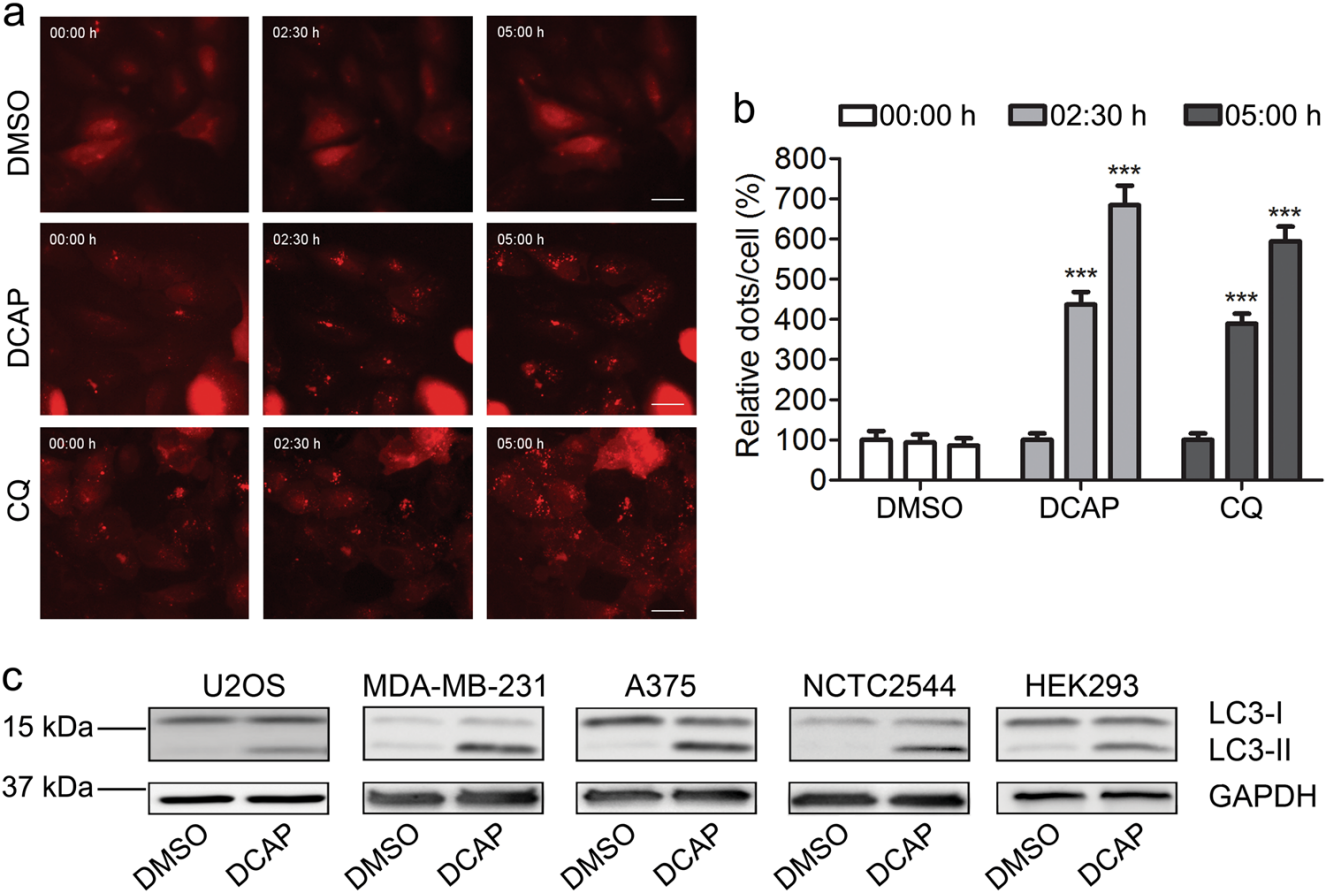
A broad-spectrum antibiotic, DCAP, modulates autophagy in human cells. **a** Human U2OS cells expressing the autophagic marker, LC3, fused with a red fluorescent protein (LC3-RFP) were treated with DMSO and equimolar concentration (50 μM) of the autophagy inhibitor, chloroquine (CQ), or the antibiotic, DCAP. Series of image frames of LC3-RFP were acquired by fluorescence microscopy at 25 min/frame over a 12 h period (Supplementary Video S1, S2 and S3). Representative sequences of images at 0, 2.5 and 5 h displaying accumulation of LC3-RFP dots overtime in DCAP and CQ-treated cells are shown. **b** Quantification of the number of fluorescent LC3-RFP dots per cell after a 2.5 and 5 h treatment with DMSO, DCAP or CQ. The number of fluorescent dots at time zero was set to 100%. Shown as mean percentage of relative dots/cell ± s.e.m., ***P < 0.001, compounds versus DMSO (two-way ANOVA with Bonferroni post tests). **c** Immunoblot analysis of unconjugated (LC3-I) and lipid-conjugated (LC3-II) form of the autophagy marker, LC3, across human bone (U2OS), mammary gland (MDA-MB-231), skin (A375, NCTC 2544) and embryonic kidney (HEK 293) cultured cells treated 24 h with vehicle (DMSO) or 10 μM DCAP. GADPH was used as a loading control

To identify novel small molecule compounds affecting autophagy, human osteosarcoma U2OS cells expressing the protein LC3 (a marker of autophagosomes) fused with a Red Fluorescent Protein (RFP-LC3) were monitored by real-time fluorescence imaging in presence or absence of selected compounds. A well-defined autophagy inhibitor, chloroquine (CQ), was adopted as a reference. U2OS RFP-LC3 cells treated with CQ showed a significant accumulation of LC3-RFP-fluorescent dots overtime, compared with vehicle (DMSO) (Fig. 1a, b and Supplementary Video S1 and S2).

With this asset, we screened a subset of small molecules present in the internal chemical collection of the Ististuto Italiano di Tecnologia (Italy), which contains both commercially available and in-house synthetized compounds. Among them, we selected ~200 diverse and non-redundant molecules with structural and chemical features different from current characterized autophagy modulators, including polyamines^24^, quinolones^25^ and cyclic-substituted amines^7,26^). This screening revealed that a carbazol-containing compound previously characterized as a broad-spectrum antibiotic, 2-((3-(3, 6-dichloro-9H-carbazol-9-yl)-2-hydroxypropyl) amino)-2-(hydroxymethyl) propane-1, 3-diol (DCAP) was a novel autophagy modulator. Indeed, DCAP stimulated the formation of RPF-LC3 puncta when added to U2OS cells (Fig. 1a, b and Supplementary Video S3).

Validating our fluorescence microscopy analysis, and extending our observation to additional human cell lines, we found that 10 µM DCAP increased the levels of endogenous lipidated LC3 (LC3-II) in cell lines from bone (U2OS), mammary gland (MDA-MB-231), skin (A375 and NCTC2544) and kidney (HEK-293) (Fig. 1c). Indicating a specific activity of DCAP toward autophagy, two additional commercially available carbazol-containing compounds were not active in our screening and did not significantly increased LC3-II protein levels in U2OS cells (Supplementary Fig. S1).

**Fig. 2:**
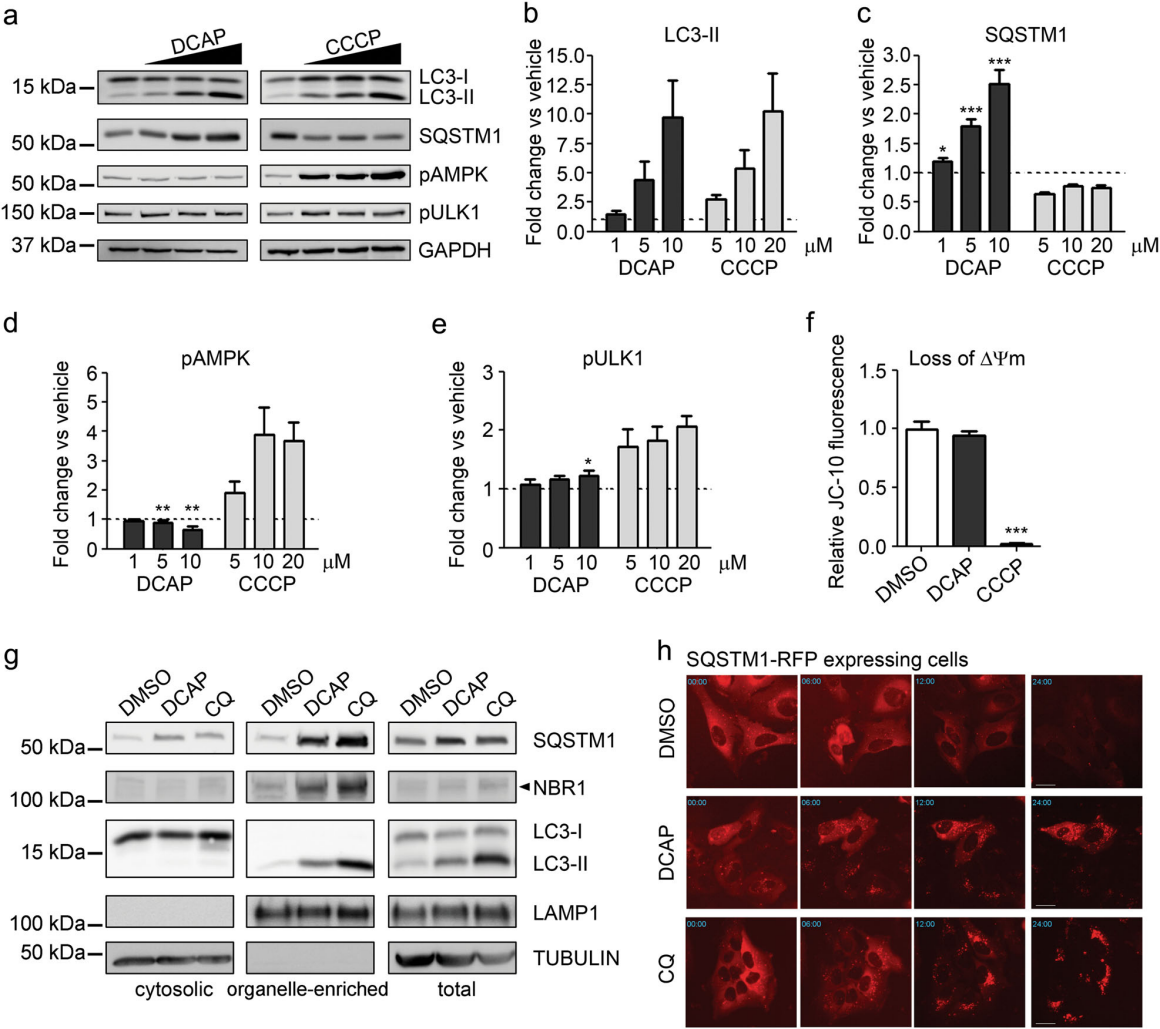
DCAP blocks the autophagic flux without impairing mitochondrial potential. **a–e** Immunoblot analysis of LC3, SQSTM1, AMPK phosphorylated in T172 (pAMPK) and ULK1 phosphorylated in S555 (pULK1) in U2OS cells treated 24 h with three increasing doses of DCAP (1, 5 and 10 μM) or CCCP (5, 10 and 20 μM). GAPDH was used as a loading control. Densitometry analysis of LC3-II (**b**), SQSTM1 (**c**), pAMPK (**d**) and pULK1 (**e**) is reported as relative protein levels normalized by GAPDH. Vehicle (DMSO) sample value was set to 1 (dotted lines in the graphs). Shown as mean ± s.e.m., *n* = 3. *P < 0.05; **P < 0.01; ***P < 0.001 DCAP versus CCCP (two-way ANOVA with Bonferroni post test). **f** Loss of mitochondrial membrane potential (ΔΨm) was quantified by measuring the amount of monomeric JC-10 dye after 24 h of treatment with vehicle (DMSO), 10 µM DCAP or 20 µM CCCP. These doses were adopted as they produced a comparable LC3-II induction (**b**). Shown as mean ± s.e.m., *n* = 6. ***P < 0.001, CCCP versus vehicle (one-way ANOVA). **g** Immunoblot analysis of cytosolic and organelles-enriched (vacuolar) preparations from cells treated 24 h with vehicle (DMSO), 10 µM DCAP or 25 µM chloroquine (CQ). Autophagic LC3-II and lysosomal LAMP1 proteins were used to confirm the enrichment in autophagosomes and autophagolysosomes in vacuolar fractions. Cytoplasmic TUBULIN protein was adopted to evaluate potential cytosolic contaminations in the organelles-enriched fraction. Immunoblot with antibodies against the autophagic receptors, SQSTM1 and NBR1, showed accumulation of these proteins in vacuolar fractions of DCAP- and CQ-treated cells. **h** Autophagosomal degradation of a SQSTM1 protein fused with a Red Fluorescent Protein (RFP) was observed by live fluorescent microscopy in SQSTM1-RFP expressing cells treated with DMSO, 10 µM DCAP or 25 µM CQ. Representative images before addition of compound (time 0) and at 6, 12, and 24 h post-treatment are shown. DCAP and CQ treatment produced accumulation of SQSTM1-fluorescent dots, overtime. Scale bar in the 24 h frame = 5 µm

DCAP was previously shown to disrupt bacterial membranes by reducing transmembrane potential of Gram-positive and Gram-negative bacteria^27^. However, high doses of DCAP also affected mitochondrial membrane potential of human cells, similar to the ionophore carbonyl cyanide m-chlorophenyl hydrazone (CCCP), whose AMPK-dependent autophagy-inducing activity is well characterized^27,28^. We thus compared the effects of DCAP and CCCP on the levels of lipid-conjugated LC3 (LC3-II), SQSTM1, and phosphorylated AMPK proteins by immunoblot analysis with specific antibodies (Fig. 2a). DCAP- and CCCP-treated cells accumulated high levels of LC3-II, thus confirming that both compounds affected autophagy (Fig. 2b). However, the two compounds generated significant opposing effects on SQSTM1 levels (Fig. 2c). While CCCP enhanced SQSTM1 autophagy-mediated degradation, thus reducing SQSTM1 signal, DCAP produced a significant accumulation of SQSTM1 in a dose-dependent manner. Furthermore, DCAP produced negligible differences in the levels of phosphorylated AMPK and its autophagy-related target, ULK1, compared with CCCP, which enhanced the signals of both phosphorylated proteins (Fig. 2d, e).

A lack of AMPK activation by DCAP indicates that this compound does not affect autophagy by impairing mitochondrial potential (ΔΨm) at the doses tested. To validate this, we assessed the ΔΨm in U2OS cells treated with a dose of DCAP and CCCP that produced a comparable increase in LC3-II levels (10 and 20 µM, respectively—Fig. 2b). Unlike CCCP, which induced a drastic loss of ΔΨm, DCAP was completely ineffective (Fig. 2f).

Our observations suggest that DCAP may block autophagy-mediated protein degradation, thus accumulating immature autophagosomes and/or autophagolysosomes. To validate this hypothesis, we prepared cytosolic and organelle-enriched fractions from cells treated with DCAP or vehicle. Because CQ enhances cellular autophagolysosome formation^7^, this compound was adopted as a reference. Immunoblot analysis with antibodies against LC3 confirmed the enrichment of autophagosomes/autophagolysosomes in the organelle fractions, which predominantly contained the membrane-bound LC3-II form (Fig. 2g). Consistent with a DCAP-mediated blockade of the autophagic flux, cells treated with this compound showed a marked accumulation of both LC3-II and SQSTM1 in organelle-enriched fractions, compared with vehicle (Fig. 2g). Furthermore, the vacuolar levels of an additional autophagic receptor, NBR1^29^, were also increased by the treatment with DCAP and CQ (Fig. 2g).

We further analyzed the levels of lipidated LC3-II in cells treated with DMSO, DCAP or CCCP in presence with the late-stage autophagy inhibitor, Bafilomycin A1 (Supplementary Fig. S2). As expected by a treatment with an autophagy inducer^30^, a co-treatment with CCCP and Bafilomycin A1 significantly increased the levels of LC3-II compared with DMSO and Bafilomycin A1 (Supplementary Fig. S2b). In marked contrast, the combination of DCAP and Bafilomycin A1 generated negligible differences compared with DMSO and Bafilomycin A1 treatment (Supplementary Fig. S2b). A lack of DCAP-mediated accumulation of membrane-bound LC3-II in presence of Bafilomycin A1 was also confirmed in organelle-enriched preparations (Supplementary Fig. S2c).

As an independent experimental validation that DCAP blocks the autophagic flux, we monitored the autophagy-mediated degradation of a SQSTM1-RFP fusion protein by time-course fluorescent microscopy. The addition of DCAP to the medium resulted in a progressive accumulation of SQSTM1-RFP punctae (Fig. 2h and Supplementary Video S4), confirming that DCAP inhibits autophagy-dependent SQSTM1 turnover.

Collectively, our data demonstrate that DCAP possesses autophagic inhibitory activity and blocks autophagic degradation.

### DCAP blocks autophagy at the last stage by impairing autophagolysosome degradation

**Fig. 3:**
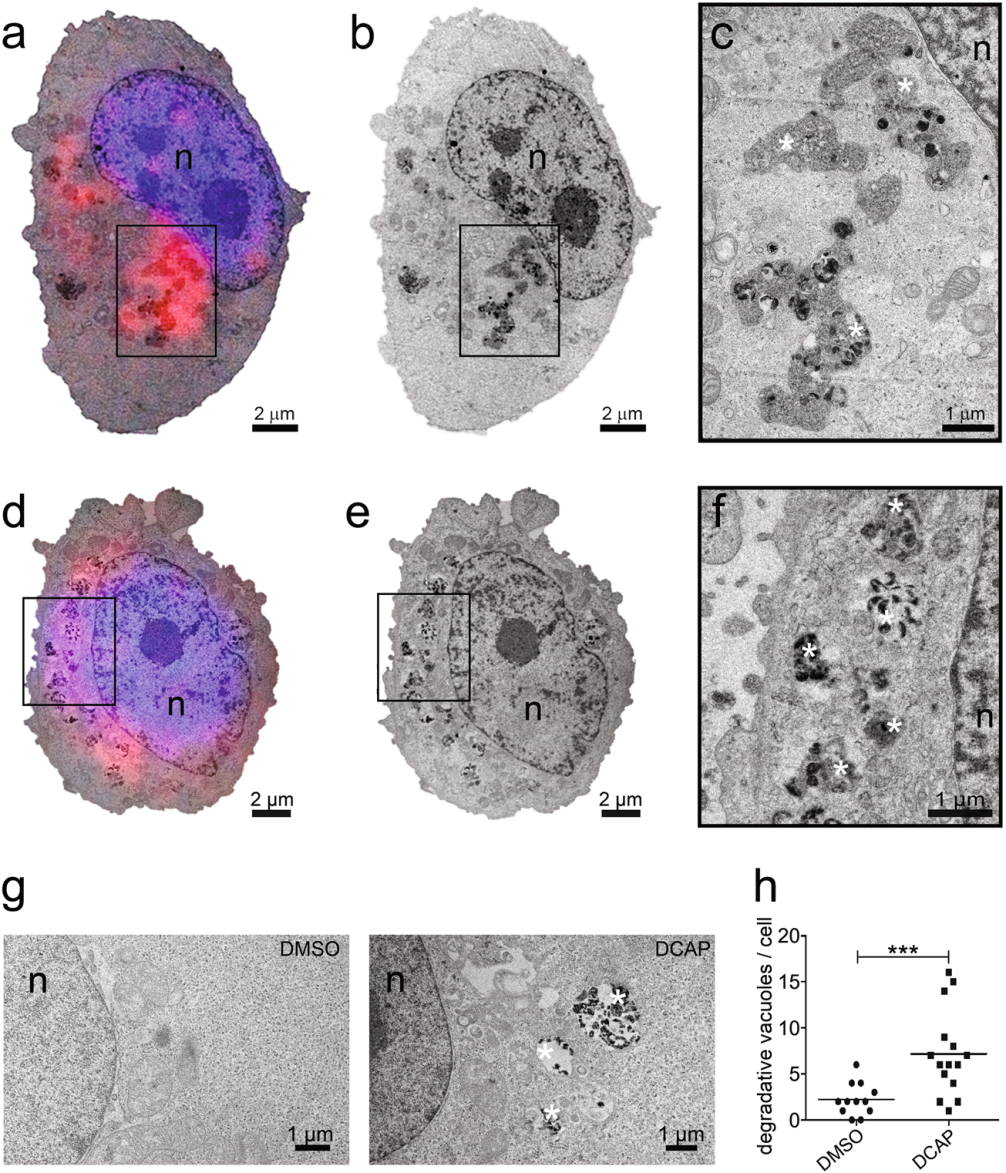
DCAP blocks autophagy at the late stage. Correlative light electron microscopy (CLEM) on SQSTM1-RFP expressing cells treated with 10 µM of DCAP for 24 h. **a**, **d** Superimposed maximal projections of z-stack confocal images and low magnification TEM slice projection image of two entire representative DCAP-treated cells. **b**, **e** TEM projection image of the same cells shown in (**a**, **d**). **c**, **f** High magnification of the region boxed in (**a**, **d**) showing a cluster of SQSTM1-fluorescent positive degradative autophagic vacuoles (AVd) containing electron dense material (dark) and membrane remnants (asterisks). **g**, **h** Quantitative assessment of degradative vacuoles in TEM sections from cells treated with DMSO or 10 µM DCAP. Example of cytoplasmic regions containing degradative vacuoles (asterisks) in DCAP-treated section is provided in **g**. Quantification in (**h**) is shown as mean degradative vacuoles per cell ± s.e.m., ***P < 0.001 DCAP versus DMSO (Mann–Whitney test)

The fact that DCAP-treated cells showed accumulation of autophagic receptors in organelle-enriched fractions (Fig. 2g) implies that this antibiotic blocks the late stage of autophagy by either preventing autophagosome/lysosome fusion or avoiding autophagolysosome maturation. To discriminate between these two diverse mechanisms, we conducted Correlative Light Electron Microscopy (CLEM) analysis on SQSTM1-RFP-expressing cells, which was grown on gridded coverslip and treated with 10 µM of DCAP for 24 h. After nuclear staining with Hoechst-33342, fluorescence images were acquired and confirmed the presence of numerous perinuclear SQSTM1-RFP dots. The samples imaged at the fluorescence microscope were next processed for TEM, obtaining ultrastructural information of the same cells analyzed by fluorescent microscopy (see representative cells in Fig. 3a, d). The CLEM analysis showed cytoplasmic accumulation of late degradative vacuoles in correspondence of the regions containing SQSTM1-fluorescent dots, characterized by a partially or completely absence of internal membranes and by the presence of degraded cytoplasmic material that appear strongly electron dense (dark) in osmium tetroxide post-fixed TEM sections^9^ (Fig. 3c, f).

The morphological feature of the observed vacuoles, together with the presence of SQSTM1 fluorescence, clearly identify these structures as late autophagolysosomes and indicates that DCAP inhibits autophagy at its last step.

In line with this concept, quantitative TEM analysis showed significant increase of degradative vacuoles in DCAP-treated cells, compared with DMSO treated cells (Fig. 3g, h).

### DCAP prevents lysosomal acidification without affecting lysosomal membrane permeabilization

**Fig. 4:**
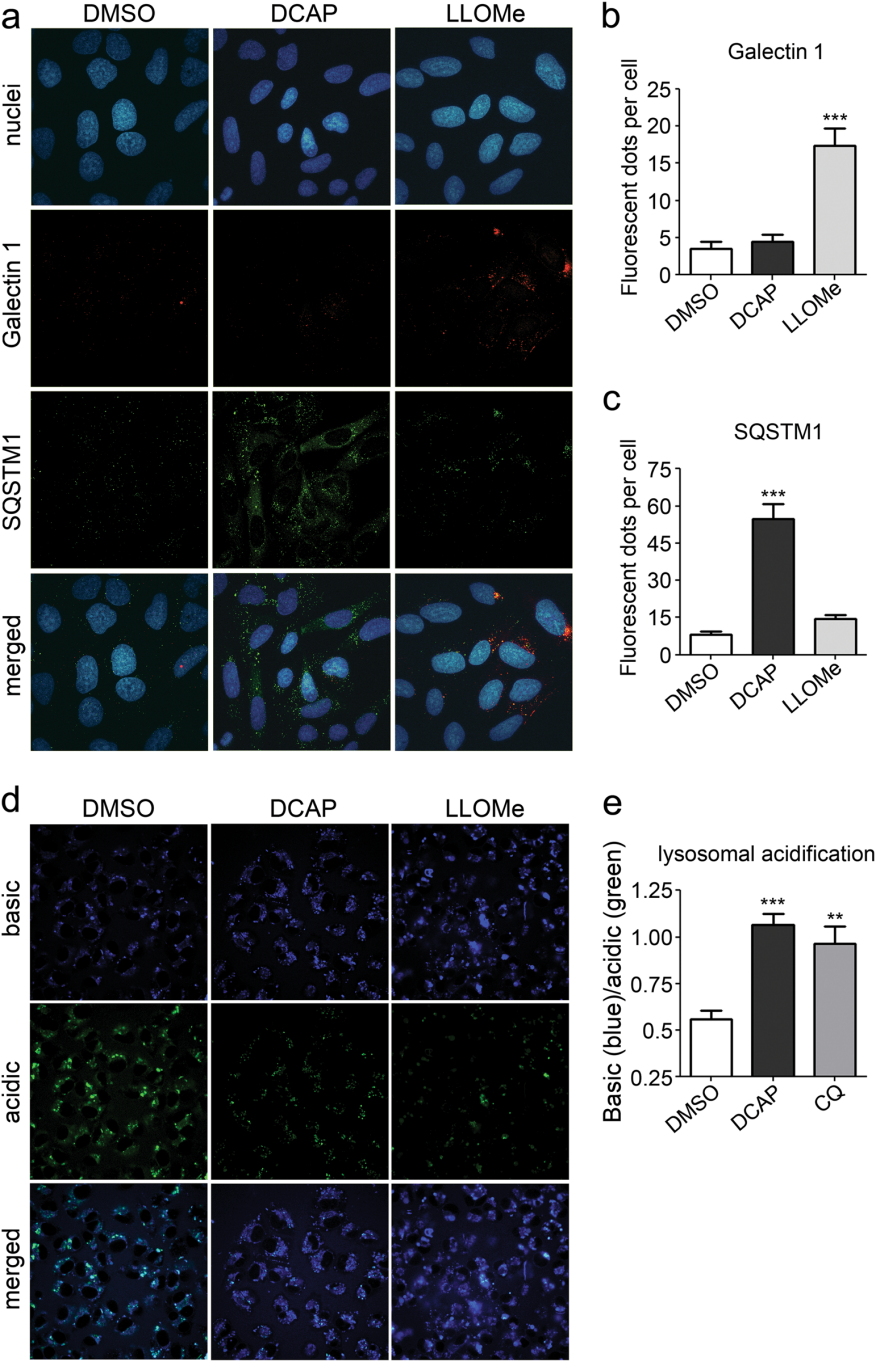
DCAP inhibits lysosome acidification without affecting lysosomal membrane permeability. **a**–**c** Lysosomal membrane permeabilization (LMP) and autophagy in cells treated with vehicle, 10 µM DCAP or L-leucyl-L-leucine methyl ester (LLOMe), which specifically induces lysosome membrane damage, was assessed by immunofluorescence microscopy with antibodies against a LMP-related marker, Galectin 1, and the autophagic receptor, SQSTM1. **a** Representative confocal images of treated cells probed with an anti-Galectin 1/ antibody/Alexa555 (red), anti-SQSTM1/Alexa488 (green) and Hoechst 33342 nuclear staining (blue). **b**, **c** Quantification of Galectin 1- and SQSTM1-positive fluorescent dots per cell. Shown as average number of fluorescent dots per cell ± s.e.m., *n* = 3. ***P < 0.001, compounds vs. DMSO (one-way ANOVA, Dunnett’s Multiple Comparison Test). **d**, **e** The effect of 10 µM DCAP or 25 µM CQ in decreasing lysosomal acidification was assessed by measuring the ratio between blue (basic) and green (acidic) fluorescent signals of a lysosomal pH indicator, LysoSensor Yellow/Blue dextran. Representative fluorescent images are shown in **d**. Quantification is shown as average basic/acidic ratio ± s.e.m, **P < 0.01 and ***P < 0.001, compounds versus DMSO (one-way ANOVA, Dunnett’s Multiple Comparison Test)

The observed accumulation of autophagolysosomal structures in DCAP-treated cells indicates that this antibiotic may impair lysosomal function. We thus assessed whether DCAP may affect lysosomal membrane stability and induce lysosomal membrane permeabilization (LMP)^31^. Several soluble carbohydrate-binding lectins, such as Galectin 1, translocate to the sites of endo-lysosomal leakage upon LMP and the evaluation of galectin puncta formation by immunofluorescence microscopy has been proven to be a specific method for LMP assessment^32^. Accordingly, we evaluated the number of Galectin 1 puncta in cells treated with DCAP or with l-leucyl-L-leucine methyl ester (LLOMe), which specifically induces lysosome membrane damage^32^. In addition, we monitored the autophagic flux by assessing the amount of SQSTM1 fluorescent dots. As expected, LLOMe-treated cells accumulated numerous Galectin 1-positive fluorescence dots (Fig. 4a, b). In marked contrast, DCAP did not significantly affected the number of Galectin 1 puncta, while it generated a significant accumulation of SQSTM1-positive dots (Fig. 4a–c).

Considering the LMP-independent autophagolysosome inhibition activity of DCAP, we determined whether this compound might block autophagy by affecting lysosomal pH, which is the mechanism of action of several known autophagy inhibitors, including CQ^33^. To this aim, we compared the ability of DCAP or CQ in affecting the ratio between the blue basic and green acidic form of a fluorescent pH indicator. The treatment with DCAP or CQ generated similar and significant increase in basic/acidic ratio of the indicator as compared with vehicle, thus showing inhibition of lysosomal acidification (Fig. 4d, e).

Thus, our data demonstrate that DCAP blocks the late stage of autophagy by inhibiting lysosomal acidification, hence preventing the final maturation/degradation step of autophagolysosomes.

### DCAP reduces autophagy-mediated UPEC infection

**Fig. 5:**
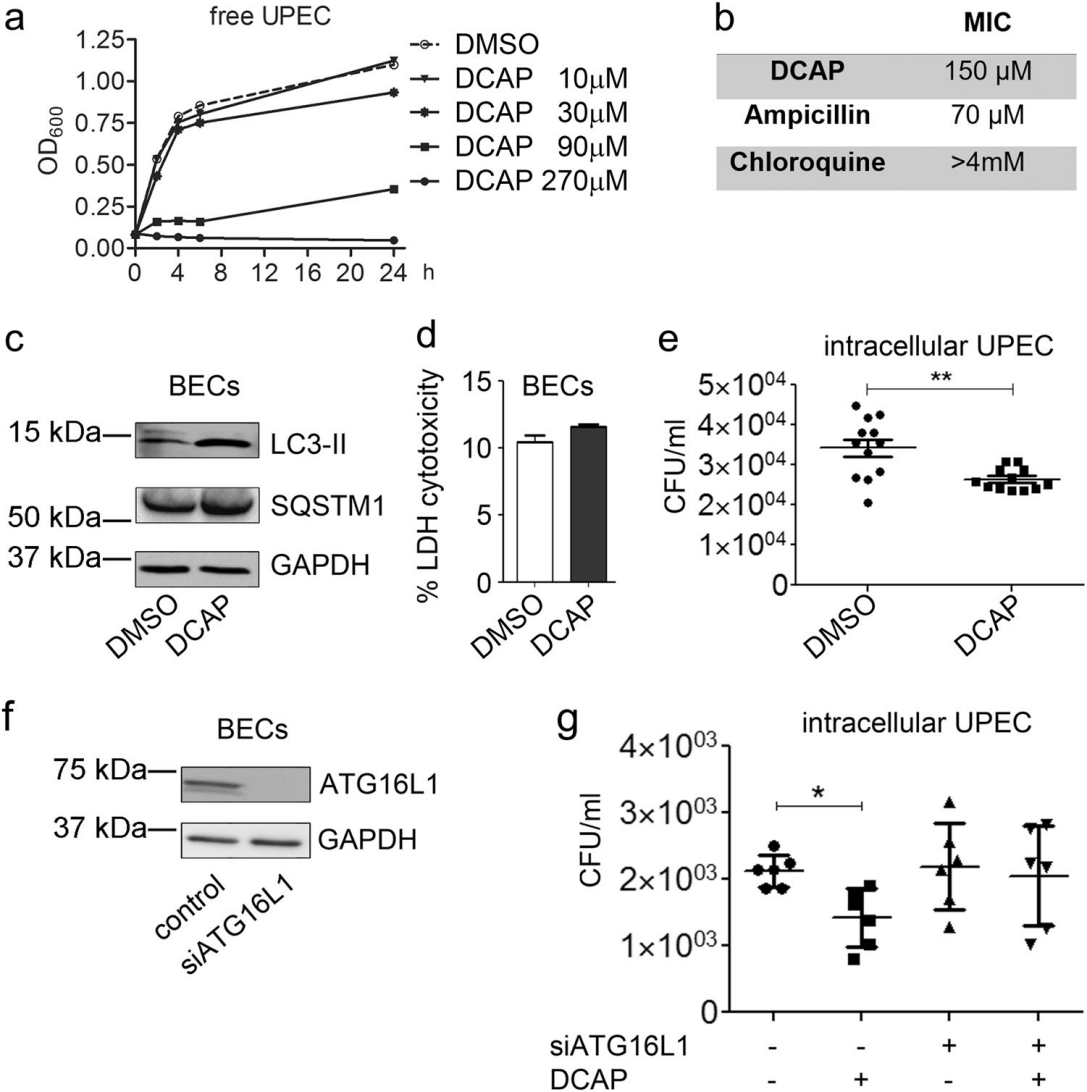
DCAP reduces autophagy-mediated uropathogenic *E.coli* infection. **a** Growth curves of free uropathogenic *E. coli* bacteria (UPEC) in presence or absence of the indicated DCAP concentrations. **b** Measurements of the minimum inhibitory concentration (MIC) of growth against UPEC bacteria of DCAP, CQ or the well-characterized beta-lactam antibiotic, ampicillin. **c** Immunoblot analysis of protein samples from Bladder Epithelial Cells (BECs) treated 24 h with vehicle (DMSO) or 10 µM DCAP, showing DCAP-mediated accumulation of LC3-II and SQSTM1. GAPDH was used as a loading control. **d** The potential cytotoxicity of 10 µM DCAP against BECs was evaluated by lactate dehydrogenase (LDH) cytotoxic assay. Indicating the tolerability of BECs toward this dose of compound, cells treated for 24 h showed negligible differences in the release of LDH in the medium, compared with vehicle-treated cells. Reported as percentage of LDH cytotoxicity ± s.e.m., *n* = 6. **e** BECs were infected with a pathogenic UTI89 *E. coli* strain. After the removal of extracellular bacteria by gentamycin, an antibiotic that is not permeable through the human cell membrane, BECs were treated with 10 µM DCAP or vehicle for 24 h. The counting of intracellular bacteria from DMSO- and DCAP-treated infected cells is reported as an average of the colony-forming unit (CFU)/ml ± s.e.m., *n* = 12. **P < 0.01 DCAP vs. DMSO (Mann–Whitney test). **f** Immunoblot analysis of protein extract from BECs transfected with a siRNA sequences against ATG16L1 or a non-targeting element (control). GAPDH was used as a loading control. **g** Quantification of intracellular UPEC bacteria in infected siATG16L1 and control BECs treated with 10 µM or DMSO. Shown as average CFU/ml ± s.e.m., *n* = 7. *P < 0.05 DCAP vs. DMSO (Mann–Whitney test)

Considering the role of autophagy in the pathogenesis of UTIs^18,19^, wherein UPEC hijack the pathway to persist in autophagosomes, the fact that a broad-spectrum antibiotic affected autophagy warranted further investigation. We inquired this possibility in an in vitro infection assay in 5637 bladder epithelial cells (BECs) infected with a pathogenic *E. coli* strain UTI89^10^. We first determined the cytotoxicity of DCAP against UTI89 cells. A dose-response treatment of free bacteria with diverse doses of DCAP revealed that this antibiotic was effective in reducing bacterial growth starting from 30 µM (Fig. 5a). Further analysis showed that the minimal inhibitory concentration (MIC) of DCAP against UTI89 pathogenic bacteria was in the low/medium micromolar range, thus comparable with the MIC of a well-characterized beta-lactam antibiotic, ampicillin (Fig. 5b). In marked contrast, the single autophagy inhibitor, CQ, was completely ineffective against bacteria at a dose as high as 4 mM (Fig. 5b).

Considering that 10 µM DCAP did not affected the growth of free UPEC bacteria (Fig. 5a), we decided to adopt this dose to specifically test a potential bactericidal-independent/autophagy-dependent activity of DCAP against intracellular bacteria. Similar to the observation in other human cell lines (Supplementary Fig. S3), 10 µM of DCAP markedly affected autophagy, but not viability, of BECs (Fig. 5c, d). Yet, this dose significantly reduced the number of intracellular bacteria in UPEC-infected BECs (Fig. 5e).

To verify that DCAP-mediated reduction of intracellular infection actually derived from a DCAP-dependent blockade of autophagy, we repeated the UPEC infection in BECs in which the expression of essential autophagy protein, ATG16L1, was silenced by siRNA (Fig. 5f). While scrambled control siRNA treated with DCAP showed a significant decrease in bacterial CFUs, the treatment of ATG16L1 silenced cells did not alter bacterial titers (Fig. 5g). Collectively, these data indicate that DCAP displays autophagy-mediated antibacterial activity.

### DCAP improves the in vitro anticancer activity of the HDAC inhibitor, vorinostat

**Fig. 6:**
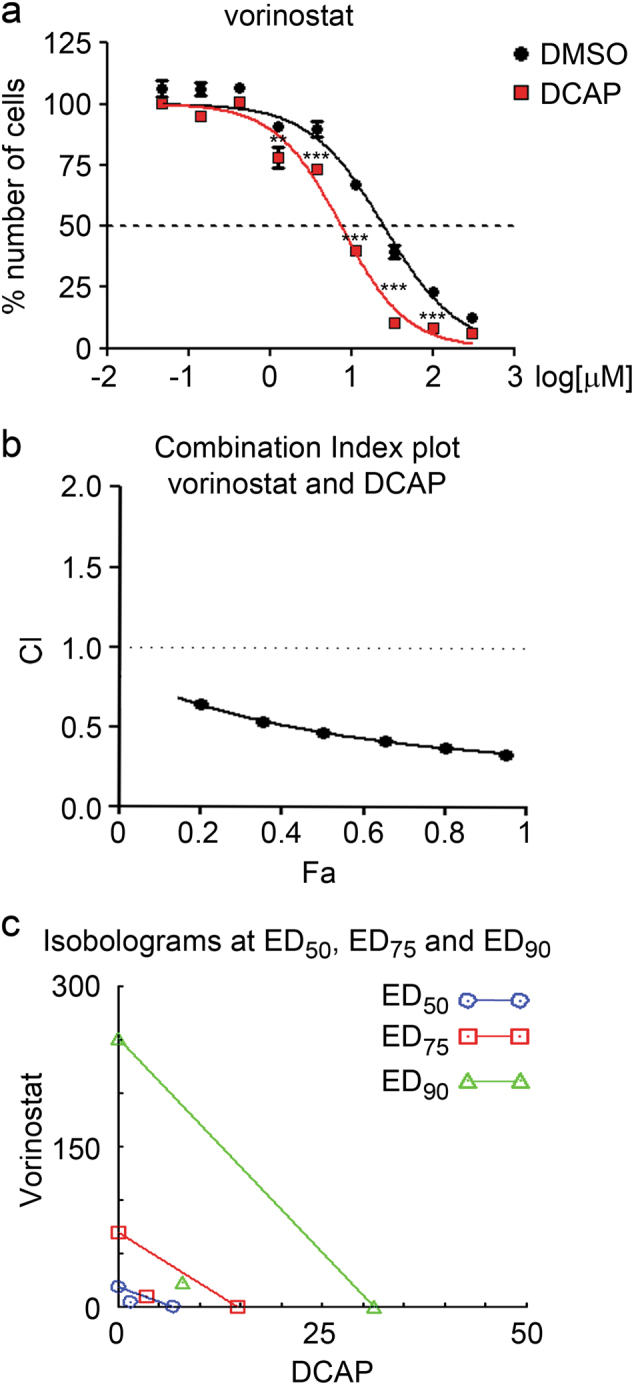
DCAP improves in vitro anticancer activity of vorinostat. **a** Concentration response plots of vorinostat cytotoxicity against breast cancer MDA-MB-231 cells in presence (red line) or absence (black line) of 5 μM DCAP for 72 h. Values of cells treated with vehicle (DMSO) were set to 100% of number of cells. Compound concentration is reported as log[μM]. The data expressed as mean ± s.e.m, *n* = 6. **P < 0.01 and ***P < 0.001, presence vs. absence of DCAP (two-way ANOVA with Bonferroni post test). **b** Combination Index plot obtained from dose-response curves of vorinostat, DCAP and a constant ratio combination of vorinostat and DCAP (3:1) in MDA-MB-231 cells. Fa = Fraction of cells Affected by the treatments. **c** Isolobograms of drug doses of vorinostat, DCAP or a combination of both compounds, required to achieve a 50% (blue), 75% (red) and 95% (green) reduction of viable cells (ED_50_, ED_75_ and ED_95_)

In vitro studies indicated that autophagy plays a protective role in vorinostat-induced cytotoxicity against cancer cells^23^. We thus evaluated the ability of DCAP in improving the anticancer activity of the HDAC inhibitor (HDACi), vorinostat, against breast cancer MDA-MB-231 cells. In presence of a sub-lethal dose of DCAP, vorinostat in vitro anticancer activity significantly improved and the concentration of this HDACi required to kill 50% of cells (IC_50_) decreased by a logarithmic factor (Fig. 6a).

We further analyzed the pharmacological interaction between DCAP and vorinostat in affecting cell viability by Chou-Talalay method for drug combination analysis^34^. Combination Index (CI) plot obtained by dose-response curves with vorinostat, DCAP and a constant ratio combination of vorinostat and DCAP (3:1) showed a synergistic interaction between the two compounds, as indicated by a CI < 1 at different Fraction of cell affected (Fa) (Fig. 6b). Further supporting vorinostat/DCAP synergy, CompuSyn generated isobologram showed a marked reduction of drug doses required to achieve a 50, 75 and 95% reduction of viable cells (ED_50_, ED_75_ and ED_95_) (Fig. 6c).

These data indicate that DCAP significantly improve the anticancer activity of the clinical relevant antineoplastic agent, vorinostat.

## Discussion

A previous screening for novel broad-spectrum antibiotic identified (2-((3-(3,6-dichloro-9H-carbazol-9-yl)-2-hydroxypropyl)amino)-2-(hydroxymethyl)propane-1,3-diol), named DCAP, as a new compound targeting the membranes of both Gram-positive and Gram-negative bacteria^27^. Accordingly, DCAP inhibited the growth of a number of clinical pathogens, including *Escherichia coli*, *Pseudomonas aeruginosa*, and *Bacillus subtilis*^27^.

Here we show that DCAP also possesses autophagy-modulating activity. Specifically, DCAP reduced the autophagic flux in a number of human cell lines from diverse tissue sources, as indicated by increased levels of the lipidated LC3 and the autophagy receptor, SQSTM1. The observed accumulation of SQSTM1 and NBR1 in autophagosome-enriched preparations can be attributed to reduced autophagic degradation upon DCAP treatment. CLEM analysis further indicated that DCAP-mediated autophagy inhibition derived from a blockade of the last step of the autophagy process. Indeed, DCAP-treated SQSTM1-RFP-expressing cells accumulated numerous autophagolysosome structures enclosing partially degraded material in the corresponding regions with elevated SQSTM1 fluorescent dots. Consistent with this, DCAP inhibited lysosomal acidification without altering lysosomal membrane properties, as indicated by the absence of Galectin 1 punctae accumulation in DCAP-treated cells.

The autophagy inhibitory activity of DCAP therefore suggests its potential use against UTIs, which are among the most frequently recurring infectious diseases in humans^35^. Since Gram-negative UPEC are known to be harbored within autophagosomes, a blockade of autophagy has been proposed as a suitable strategy for reducing UPEC reservoirs^18^. In line with this, UPEC-infected bladder epithelial cells treated with DCAP showed a significant reduction in intracellular bacterial persistence. Furthermore, DCAP treatment did not affect bacterial persistence in epithelial cells lacking the essential autophagy gene, ATG16L1.

The clinically relevant autophagy inhibitor, CQ, has been also shown to reduce intracellular UPEC bacteria, by blocking autophaglysosome maturation^10^. Nonetheless, CQ presents a reduction in neutrophil count (neutropenia) as a common adverse effect^36,37^. Because neutrophils comprise a fundamental protective defense mechanism against pathogens, drug-related neutropenia highly increases the susceptibility to both Gram-positive and Gram-negative bacterial infections^38^. In this context, CQ presents a high liability for its use in antibacterial treatment due to the risk of generating novel bacterial infection. Therefore, compounds with dual inhibitory activity against bacteria and autophagy, such as DCAP, may be a valuable alternative.

Notably, we further showed that DCAP effectively potentiates the anticancer activity of the clinical relevant antineoplastic agent, vorinostat, which similar to CQ presents neutropenia as a common adverse effect^39^. Accordingly, the use of DCAP or DCAP-derived analogs as adjuvant therapy in vorinostat treatment may be a suitable approach to enhance anticancer activity while preventing a chemotherapy-related increased risk of bacterial infections.

Moreover, compared with a combination of single antibacterial and autophagy inhibitory drugs, a molecule having a multiple activity toward autophagy and bacteria would present additional polypharmacology-related advantages, including more predictive pharmacokinetics and reduced risk of drug interactions^40^.

Future medicinal chemistry efforts can be now attempted to improve DCAP biological activity toward bacteria and autophagy, and to evaluate/optimize the drug-like properties of this class of compounds. In this context, a preliminary structure activity relationship (SAR) exploration around DCAP has elucidated some chemical and structural features associated with its antibacterial potency^41^. In particular, the DCAP analog, 1-(3,6-dichloro-9H-carbazol-9-yl)-3-[(3-methylbutyl)amino]propan-2-ol (**10**), inhibited the growth of an *E. coli* strain at low micromolar doses^41^. Furthermore, analog **10** showed synergistic activity with kanamycin^41^, indicating potential combinatorial use of these antibiotics against UPEC.

The newly identified autophagy inhibition activity of DCAP permits the exploration of whether DCAP analogs with improved antibacterial potency will be still effective in blocking autophagy. Alternatively, these studies will provide important information on the structural and chemical features associated with DCAP-mediated autophagy inhibition, allowing further chemical optimization to improve the bactericidal activity while preserving the autophagy inhibitory feature.

In conclusion, our data identify DCAP as a novel multi-target molecule acting on both autophagy and bacteria, and support the future development of this compound for the delivery of bactericidal autophagy blockers for uropathogenic infections and for antibacterial adjuvant therapy in cancer treatment.

## Materials and Methods

### Chemicals

2-((3-(3,6-dichloro-9H-carbazol-9-yl)-2-hydroxypropyl)amino)-2-(hydroxymethyl)propane-1,3-diol (DCAP) (Sigma, SML0515-5MG), Carbonyl Cyanide 3-ChloroPhenylhydrazone (CCCP) (Sigma, C2759), H-Leu-Leu-OMe hydrochloride (LLOMe) (Santa Cruz, sc-285992), vorinostat (Sigma, SML0061), chloroquine (Sigma, C6628), bafilomycin A1 (Sigma, B1793), digitonin (Sigma, D141). All compounds were dissolved in dimethyl sulfoxide (DMSO) and a “compound-free” DMSO solution as a comparison (vehicle) was adopted to rule out potential compound-independent biological activity related with the DMSO.

### Cell culture

Human osteosarcoma cells U2OS, human embryonic kidney cells 293 (HEK293), human melanoma cancer cells A375 and human urinary bladder epithelial carcinoma cells (5637, HTB9) were acquired from American Type Culture Collection and the National Collection of Type Cultures (ATCC), were grown in Dulbecco’s modified Eagle’s medium (DMEM, Euroclone, ECB7501L) with 4.5 g/L glucose supplemented with 10% fetal bovine serum (FBS, Gibco, 10270106), 2 mM L-glutamine and 1% Penicillin/streptomycin. Breast cancer cells, MDA-MB-231, were cultured in DMEM with 4.5 g/L glucose supplemented with 5% FBS, 4mM L-glutamine and 1% Penicillin/streptomycin. Bladder carcinoma cells, 5637, and human keratinocyte NCTC2544 were cultured in RPMI 1640 medium (Euroclone, ECB9006L) supplemented with 10% FBS and 2 mM L-glutamine. All cell lines were grown at 37 °C in a humidified atmosphere with 5% CO_2_.

### Live fluorescence microscopy

The Premo™ Autophagy Sensor RFP-p62 Kit and Premo™ Autophagy Sensor LC3B-RFP (BacMam 2.0) (Invitrogen™, P36241 and P36236) were used to overexpress the autophagic marker p62 and LC3 according to company instruction. Briefly, cells were seeded on 48-well plates with bottom glass (Mattek Corporation, P48G-1.5-6-F) and the day after infected with viruses expressing p62-RFP or LC3-RFP. Forty-eight hours later, cells were treated and recorded with a fluorescent inverted microscope Eclipse Ti-E (Nikon), with an Okolab incubation system to maintain a humidified atmosphere, 37 °C and 5% CO_2_ during the duration of the experiment.

### Immunoblot analysis

Proteins were extracted by scraping cells in presence of Radio Immunoprecipitation Assay Buffer (RIPA) and protein concentration quantified with BCA assay (Euroclone, EMP014500). Western blot analyses were performed on 20 µg of protein extracts, added with 4× of Laemmli Buffer + 100 mM DTT (Biorad, 161-0747) and denatured at 95 °C for 5 min. Samples were run on 4–15% polyacrylamide gels at 90–130 V and then transferred onto nitrocellulose membrane (GeHealthcare Life Science, 10600001) for 2 h at 100 V and 1.5 A of maximum electric current intensity. After blocking in 5% non-fat milk in TBS-Tween 20 0,1% (TBST), membranes were probed overnight at 4 °C with anti-LC3 primary antibody (1: 2000, MBL), anti-SQSTM1 (1:2000, Santa Cruz), anti-NBR1 (1:1000, Cell Signalling), anti-pULK1 s555 (1:1000, Cell Signalling), anti-LAMP1 (1:1000, cell Signalling), anti-GAPDH (1:1000, Invitrogen), anti-Actin (1:100 000, A1978), anti-Tubulin (Cell Signalling, 2128S). Next day, membranes were incubated with horseradish peroxidase-conjugated secondary antibody (goat-anti-rabbit or goat-anti-mouse, Millipore) at room temperature for 1 h. Proteins were visualized by the chemiluminescent substrate, ECL Star (Euroclone, EMP001005), using the ImageQuant LAS-4000 Chemiluminescence and Fluorescence Imaging System (Fujitsu Life Science). Densitometry analysis was carried out using ImageJ software 1.51n (Wayne Rasband, National Institutes of Health).

### Autophagosome-enriched preparation

Autophagosome-enriched preparation has been performed as described previously^42,43^. Briefly, cells treated with DCAP or chloroquine (CQ) were collected using trypsin and collected by low speed centrifugation. Packed cell pellets were resuspended in phosphate-buffered saline, PBS, (Sigma-Aldrich, D8537) containing 100 μg/ml of digitonin, then cytosolic and organelle-bound proteins were separated by centrifugation at 7000 r.p.m. for 8 min. Organelle-enriched pellets were solubilized in RIPA buffer, mixed with Laemmli buffer and DTT and boiled as reported above.

### Measurement of mitochondrial membrane potential

Mitochondrial membrane potential was evaluated using the Mitochondrial Membrane Potential Kit (MAK159, Sigma), according to manufacturer instructions. Briefly, cell were seeded in black 96-well culture plates with clear bottom (Perkin Elmer); after 24 h of treatment with DCAP 10 μM or CCCP 20 µM, cells were stained 1 h at 37 °C with JC-10 Dye and then read with Tecan Wallac microplate reader. Ratio 525 nm/590 nm was used to evaluate membrane potential of treated samples over control.

### Lysosomal membrane permeabilization assay

For lysosomal membrane permeabilization assay, cells were grown on coverslips and treated for 24 h with 10 μM DCAP or 25 μM CQ. A treatment with 2 mM LLoME for 2 h was used as a control^32^. Samples were then fixed with methanol for 10 min, washed twice with PBS, permeabilized with 0.1% Triton X and blocked with 3% BSA. Cells staining was performed separately with Galectin 1 antibody (1:1000 in BSA 3%, Abcam, ab25138) and SQSTM1 (Santa Cruz, B0316) overnight at 4 °C; matching Alexa Fluor 488- or 555-coupled secondary antibodies (Invitrogen, A11029 and A-21429) were probed 1 h at room temperature with 1:200 dilutions. Images were acquired with A1R+/ A1+ confocal laser microscope system Nikon and quantified using ImageJ software 1.51n (Wayne Rasband, National Institutes of Health).

### Lysosomal pH determination

The LysoSensor yellow/blue dextran Kit (Invitrogen, L22460) was used to investigate lysosomal acidification alteration. Cells were loaded with 0.5 mg/ml of dye and treated with DCAP or CQ. After 24 h, cells media were replaced with medium without phenol red and dye. Fluorescence was observed with A1R+/ A1+ confocal laser microscope system Nikon. Since the LysoSensor Dye exhibit a dual emission, pH dependent-fluorescence, ratio of blue over green fluorescence was quantified using ImageJ software 1.51n (Wayne Rasband, National Institutes of Health).

### Transmission electron microscopy (TEM) and correlative light electron microscopy (CLEM)

SQSTM1-RFP expressing cells grown on photoetched gridded coverslips (Bellco Glass Inc., USA) and treated with 10 µM DCAP for 24 h. Live cell fluorescence were then imaged using a laser scanning confocal microscope equipped with a resonant scanner (Nikon A1R) and further processed for TEM as previously described^7^. Briefly, the embedded cells were released from the coverslip by transferring the sample between liquid nitrogen and hot water. Sections of about 70 nm were cut with a Diatome diamond knife on a Leica EM UC6 ultramicrotome. TEM images were obtained with a JEOL JEM 1011 Transmission Electron Microscope operating at 100 kV of acceleration voltage and recorded with a 2 Mp charge-coupled device (CCD) camera (Gatan Orius SC100). Quantifications of degradative vacuoles was performed on more than 15 cells/samples on TEM processed samples, as previously described^44^.

### In vitro UPEC infections

In vitro infection assay was performed as described elsewhere^10^. Briefly, a clinical UPEC isolate, UTI89, was grown statically for 17 h in Luria-Bertani (LB) broth at 37 °C prior to infection of cells. Confluent 5637 cells were challenged with UPEC. After bacteria were added, plates were centrifuged at 120 × *g* for 5 min, and then incubated for 1 h at 37 °C. Extracellular bacteria were then removed by washing twice with PBS, and medium containing 0.1 mg/mL gentamicin was added to remove extracellular bacteria. 5637 cells were then incubated in gentamicin-containing medium in presence of DCAP for an additional 23 h (referred to as 24 h post infection [hpi]). Next day, 5637 cells were washed and treated with 0.1% Triton-X 100 to release bacteria. Serial dilutions of bacteria were plated on LB agar, and colony-forming units were counted.

### Cytotoxicity assay

Cell counting was performed with Countess II Automated Cell Counter (Thermofisher) in presence of Trypan blue dye to discriminate live and death cells. Proliferation and viability was assessed by Cyquant assay (Invitrogen) as previously described^7^. For drug interaction analysis, combination Index (CI) plot and isobologram were generated by Compusyn software^34^.

### Statistical analysis

Log(inhibitor)-versus-response curves and statistical analysis tests were conducted with GraphPad Prism software (San Diego, CA, USA). A *p* value < 0.05 was considered significant.

## Electronic supplementary material

Supplementary Video S1. Real time fluorescence imaging of LC3-RFP distribution within cells in DMSO treated-cells

Supplementary Video S2. Real time fluorescence imaging of LC3-RFP distribution within cells in CQ-treated cells

Supplementary Video S3. Real time fluorescence imaging of LC3-RFP distribution within cells in DCAP-treated cells

Supplementary Video S4. Real time fluorescence imaging of SQSTM1-RFP accumulation within DCAP-treated cells

Figure S1. The carbazol-containing antibiotic compound, DCAP, modulates autophagy

Figure S2. Evaluation of DCAP-mediated effect on autophagy in presence of Bafilomycin A1

Figure S3. Effect of DCAP on human cell viability
