# Supplementary figures and images for: A broad-spectrum antibiotic, DCAP, reduces uropathogenic Escherichia coli infection and enhances vorinostat anticancer activity by modulating autophagy
Source: Cell Death Dis. 2018 Jul 13;9(7):780. doi: 10.1038/s41419-018-0786-4 (PMC6045594; doi:10.1038/s41419-018-0786-4)

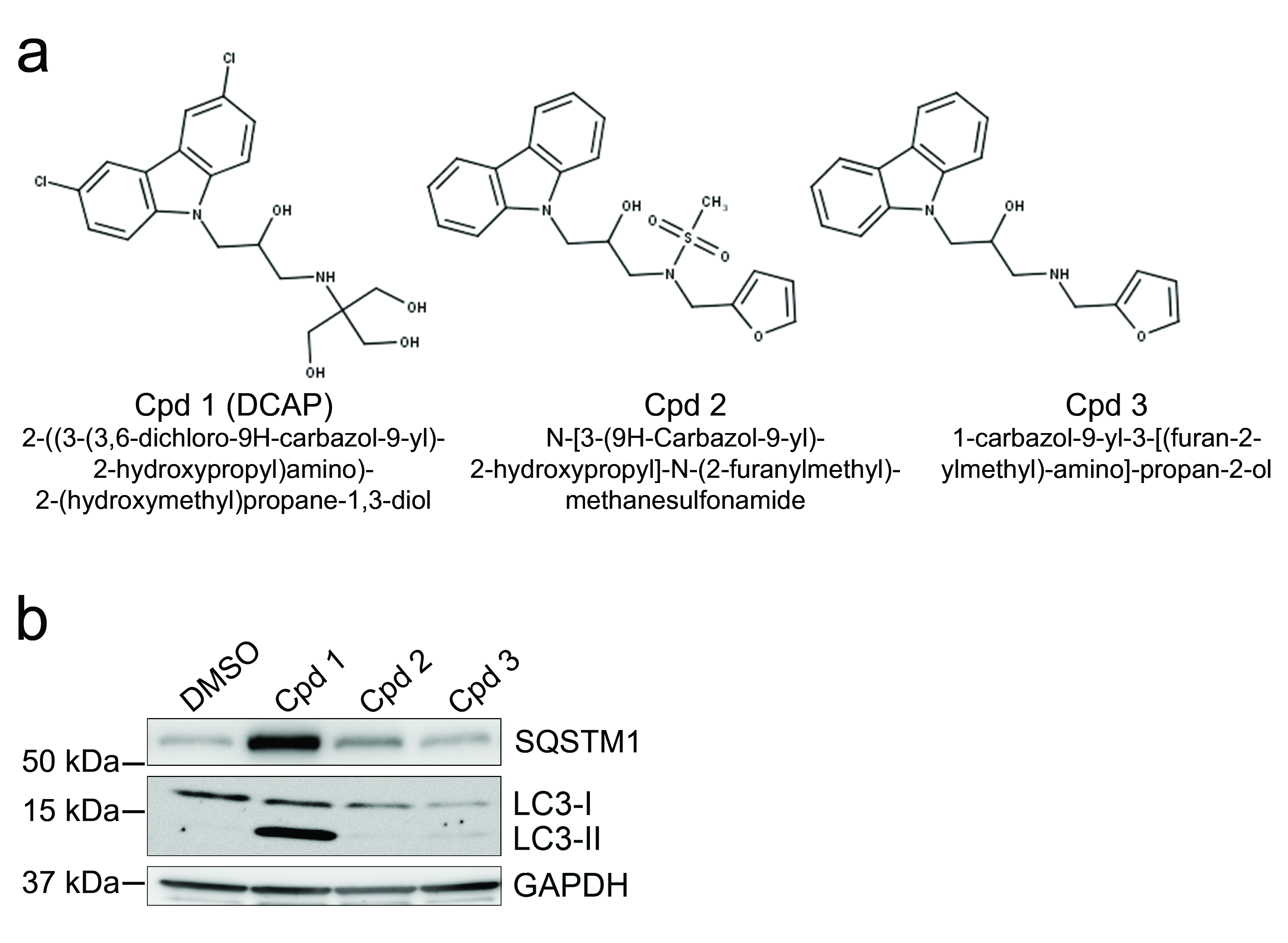

Supplement: Supplementary file 5 — Figure S1. The carbazol-containing antibiotic compound, DCAP, modulates autophagy [file 41419_2018_786_MOESM5_ESM.tif]

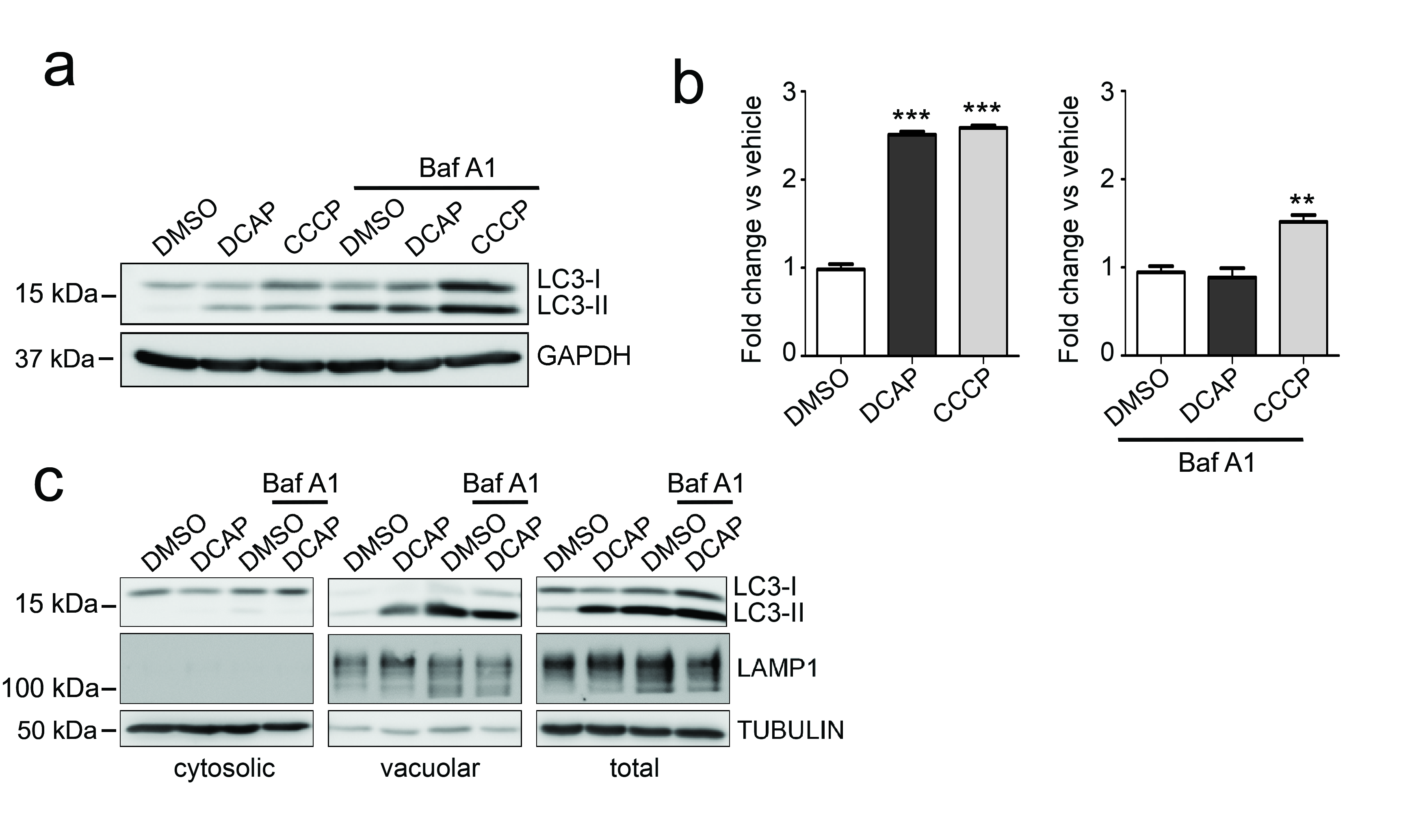

Supplement: Supplementary file 6 — Figure S2. Evaluation of DCAP-mediated effect on autophagy in presence of Bafilomycin A1 [file 41419_2018_786_MOESM6_ESM.tif]

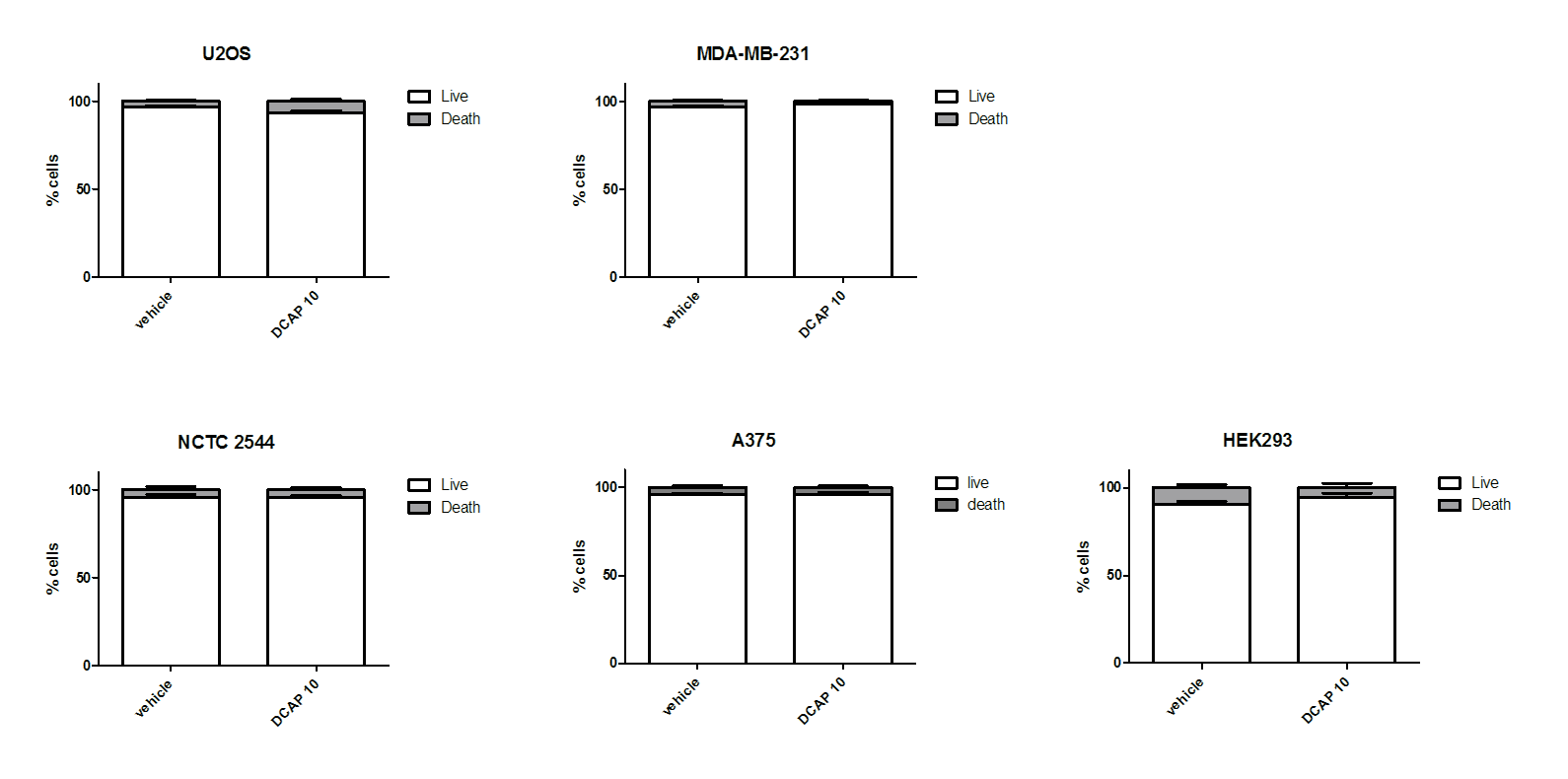

Supplement: Supplementary file 7 — Figure S3. Effect of DCAP on human cell viability [file 41419_2018_786_MOESM7_ESM.tif]
